# A gate-tunable symmetric bipolar junction transistor fabricated *via* femtosecond laser processing†

**DOI:** 10.1039/d0na00201a

**Published:** 2020-03-18

**Authors:** Bao-Wang Su, Bin-Wei Yao, Xi-Lin Zhang, Kai-Xuan Huang, De-Kang Li, Hao-Wei Guo, Xiao-Kuan Li, Xu-Dong Chen, Zhi-Bo Liu, Jian-Guo Tian

**Affiliations:** The Key Laboratory of Weak Light Nonlinear Photonics, Ministry of Education, Teda Applied Physics Institute and School of Physics, Nankai University Tianjin 300071 China liuzb@nankai.edu.cn; Institute for New Energy Materials and Low Carbon Technologies, School of Materials Science and Engineering, Tianjin University of Technology Tianjin 30071 China; Renewable Energy Conversion and Storage Center, Nankai University Tianjin 300071 China; The Collaborative Innovation Center of Extreme Optics, Shanxi University Taiyuan Shanxi 030006 China

## Abstract

Two-dimensional (2D) bipolar junction transistors (BJTs) with van der Waals heterostructures play an important role in the development of future nanoelectronics. Herein, a convenient method is introduced for fabricating a symmetric bipolar junction transistor (SBJT), constructed from black phosphorus and MoS_2_, with femtosecond laser processing. This SBJT exhibits good bidirectional current amplification owing to its symmetric structure. We placed a top gate on one side of the SBJT to change the difference in the major carrier concentration between the emitter and collector in order to further investigate the effects of electrostatic doping on the device performance. The SBJT can also act as a gate-tunable phototransistor with good photodetectivity and photocurrent gain of *β* = ∼21. Scanning photocurrent images were used to determine the mechanism governing photocurrent amplification in the phototransistor. These results promote the development of the applications of multifunctional nanoelectronics based on 2D materials.

## Abstract 2

A bipolar junction transistor with good amplification output and gate tunable photocurrent gain has been conveniently fabricated by femtosecond laser processing.

## Graphical abstract

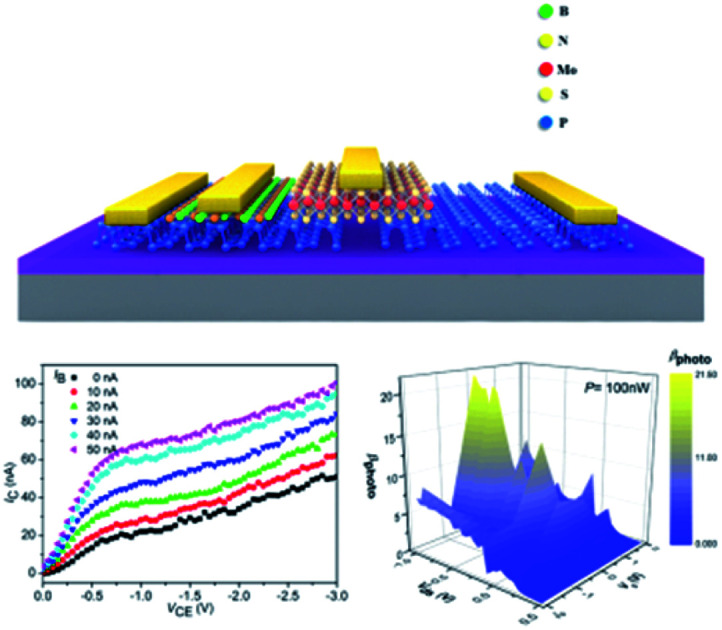

## Introduction

The development of microelectronics technology is inseparable from the 1951 invention of bipolar junction transistors (BJTs), which has helped to produce the digital revolution over the past half-century.^1,2^ A BJT, as a three-terminal (emitter, base and collector) device, is the fundamental building block of modern electronic devices, and its main feature is signal gain.^3^ These devices have been used in high-power amplifiers, high-frequency switches, analog circuits, and radio frequency (RF) systems,^4–6^ making them widely used in consumer electronics (*e.g.*, communication products), computers, audio-visual systems, and sound equipment.^7^ Besides, low power BJTs have also been widely used for the small-signal applications of amplifiers, controllers, oscillators and switches, such as electronic ballasts and mobile phone chargers.^8^ With the progress of the modern industry and the development of nanotechnology, the applications of nano-dimensional integrated high-performance devices have become the trend of future electronics. As a result, BJTs based on traditional bulk materials, including silicon/silicon–germanium alloys, aluminum gallium arsenide/gallium arsenide, and indium phosphide/indium gallium arsenide,^9–13^ will be not able to meet the demand due to their difficult fabrication procedures and limited performance.

Two-dimensional (2D) materials, including graphene,^14,15^ semiconductors (*e.g.*, transition metal dichalcogenides (TMDs),^16,17^ and black phosphorus (BP)^18^), and insulators (*e.g.*, hexagonal boron nitride (hBN)^19^) have been extensively studied due to their splendid electrical, thermal, optoelectrical, and mechanical properties.^20–24^ One of the most significant investigations in 2D materials is in van der Waals heterostructure devices.^14^ Multifunctional p–n diodes,^25,26^ ultrasensitive photodetectors,^27,28^ high-performance memories,^29,30^ light-emitting diodes,^31^ and bipolar junction transistors^32^ have been fabricated from these materials, showing their potential application in future nanoelectronics. Although several studies on BJTs based on 2D van der Waals heterostructures can be found in the literature,^33–38^ these structures have an intricate growth procedure^33,34^ and tedious multistep transfer process,^35–37^ making them difficult to fabricate. More importantly, one of the critical factors that influences BJT performance is the difference in the major carrier concentration between the emitter and collector, which has never been explored until now. A BJT can also operate as a phototransistor (where the base is left floating).^33^ Photo FETs based on other TMDs have shown large responsivity and gains, which are mainly operated in photoconductive mode or photovoltaic mode. However, the mechanism of photo-induced current gain in bipolar phototransistors is different from those photodetectors or phototransistors based on two-dimensional materials. In a bipolar phototransistor, the inherent photodiode produces a photocurrent that is subsequently amplified by the internal electrical gain in the BJT. As such, bipolar junction phototransistors could present ultrasensitive photoelectric response in weak light intensity scenarios, due to their additional internal gain, to amplify the weak photoinduced current. Besides, the bipolar transistors can also achieve well-rounded performance in terms of gain, bandwidth, responsivity, quantum efficiency, and dark current noise.^39–42^ Therefore, it is significant to fabricate high-performance van der Waals heterostructure BJTs in a convenient way.

In this paper, we present a PNP symmetric bipolar junction transistor (SBJT) fabricated with p-type black phosphorus (BP) and n-type MoS_2_ with femtosecond laser processing (FSLP).^43–45^ Compared with other intricate growth procedures and tedious transfer processes, we can produce devices with a single stacking step using FSLP. First, this SBJT exhibits a p–n junction rectification ratio of 10^3^ and photoresponsivity of 2.2 A W^−1^. This SBJT exhibits bidirectional electrical amplification output thanks to its symmetric energy band structure since the emitter and collector have the same thickness and crystal orientation. We placed a top gate on one side of the SBJT to change the difference in the major carrier concentration between the emitter and collector in order to further investigate the effect of electrostatic doping on the device's performance. Moreover, this SBJT also acts as a kind of phototransistor and exhibits maximum photoresponsivity of *R* = 151 mA W^−1^ and maximum photocurrent gain of *β* ∼ 21. Scanning photocurrent images (SPI) were used to determine the mechanism governing photocurrent amplification in the SBJT, which was reported in our previous studies.^45–47^ These results illustrate a novel, convenient method for fabricating multifunctional heterostructure devices.

## Results and discussion

**Fig. 1:**
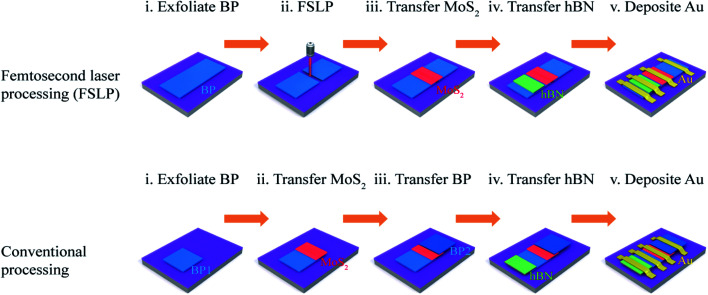
Schematic of the fabrication procedure for femtosecond laser processing (FSLP) and conventional processing.

The fabrication procedure for a BP/MoS_2_/BP SBJT with femtosecond laser processing (FSLP) and conventional processing is schematically illustrated in Fig. 1. First, a thin BP flake of uniform thickness was mechanically exfoliated using adhesive tape from a bulk BP crystal and placed on a 285 nm SiO_2_/p^+^-doped Si substrate. We used FSLP (800 nm, 35 fs, and 30 mW) to cut the BP into two pieces. More details about FSLP are presented in Fig. S1.† Next, the MoS_2_ flake was used to bridge the two BP pieces using a dry-transfer technique, which was reported in our previous studies.^45–47^ Subsequently, the hBN flake was transferred onto the left BP flake in the same way as the top gate dielectric. Finally, 50 nm thick Au electrodes were patterned and deposited using photolithography and magnetron sputtering, respectively. Fig. S2† shows optical microscope images of the SBJT after each fabrication step.

In contrast to FSLP, conventional processing is more complicated and difficult. This method not only includes multistep transfer but also needs to guarantee that the top BP sample cannot contact the bottom BP sample during the transfer. In particular, it is hard to keep the thickness and crystal orientation of the top BP sample consistent with that of the bottom BP sample. Thus, it becomes unpractical to further investigate the effect of electrostatic doping (*i.e.* the difference in the major carrier concentration between the emitter and collector) on the device's performance.

**Fig. 2:**
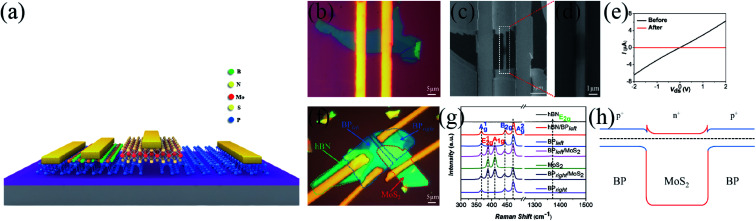
Characterization of the BP/MoS_2_/BP SBJT. (a) Schematic of the SBJT. (b) Optical microscope image of the BP device before FSLP. (c) and (d) SEM images of the region after FSLP. (e) *I*–*V* curves from the BP device before (black) and after (red) FSLP. (f) Optical microscope image of the SBJT. The left and right BP flakes are outlined in blue, MoS_2_ is outlined in red, and hBN is outlined in green. (g) Raman spectra of BP, MoS_2_, hBN flakes and heterojunctions. (h) Band structure in (p-type) BP/(n-type) MoS_2_/(p-type) BP SBJT.

Fig. 2a shows a schematic of the SBJT. To ensure that our FSLP can completely separate a BP flake, we first fabricated an individual BP device to investigate its optical and electrical properties before and after FSLP. Fig. 2b shows an optical microscope image of the BP device before FSLP. The scanning electron microscope (SEM) images in Fig. 2c and d show that the minimum linewidth can be reduced to 1 μm, which is confined to our facility and process approach, and we can clearly see that the central region of the BP sample vanished after FSLP. Fig. 2e shows *I*–*V* curves for such a device. Before FSLP, the device exhibited typical ohmic contact behavior (black line). After FSLP, the device was no longer conductive (red line), indicating that the pristine BP flake was cut into two pieces. An optical microscope image of the SBJT is shown in Fig. 2f. The left and right BP flakes are outlined in blue, MoS_2_ is outlined in red, and hBN is outlined in green. The thickness of the BP, MoS_2_, and hBN flake, were measured to be ∼7, ∼13, and ∼14 nm, respectively, using atomic force microscopy measurements, as presented in Fig. S3.† We can also see that the BP flake was well preserved and there were no obvious oxidized spots. In addition, considering the thickness dependence of MoS_2_ (base) for the performance of BJT, there are two reasons for choosing this kind of MoS_2_ flake as the base layer. First, the thinner base layer is susceptible to electrical breakdown with higher input current or bias voltages; besides, when the device operates in the forward active region, the collector current *I*_C_ would better remain constant. If the base is too thin, the holes from the emitter may overcome the barrier height and contribute to *I*_C_. Second, if the base layer is too thick, which means the increase in the concentration of the majority carrier (electrons) in the base layer, this results in more carriers (injected from the emitter) being recombined with the carriers of opposite polarity in the base layer so that the current amplification gain will be decreased. The similar thicknesses of BP, MoS_2_ and hBN flakes were used for another sample. Fig. 2g shows the Raman spectra from BP, MoS_2_, the hBN flake, and the heterojunction. Raman peaks observed at ∼361 cm^−1^, ∼439 cm^−1^, and ∼466 cm^−1^ correspond to Ag^1^, B_2g_, and A^2^_g_ phonon modes in BP,^35,47^ respectively. The peaks observed at ∼383 cm^−1^ and ∼408 cm^−1^ correspond to the E^1^_2g_ and A_1g_ phonon modes of MoS_2_, respectively,^35,36^ and the peaks observed at ∼1366 cm^−1^ correspond to the E_2g_ phonon modes in hBN.^19^ Raman modes from the overlapped region of the stacked hBN/BP (left), BP (left)/MoS_2_, and BP (right)/MoS_2_ layers correspond to peaks for each flake, which indicates good film quality in the junction region after exfoliation and dry-transfer. The band structure of the (p-type) BP/(n-type) MoS_2_/(p-type) BP SBJT are shown in Fig. 2h. The left and right BP regions have the same thickness and crystal orientation, and the major carrier concentration (holes) and band gap in the left BP are the same as those in the right BP, forming a symmetric structure. On this basis, we placed the hBN as the top gate dielectric to modulate the hole concentration in the left BP and break the symmetry and further study the effect of electrostatic doping on the device's performance.

**Fig. 3:**
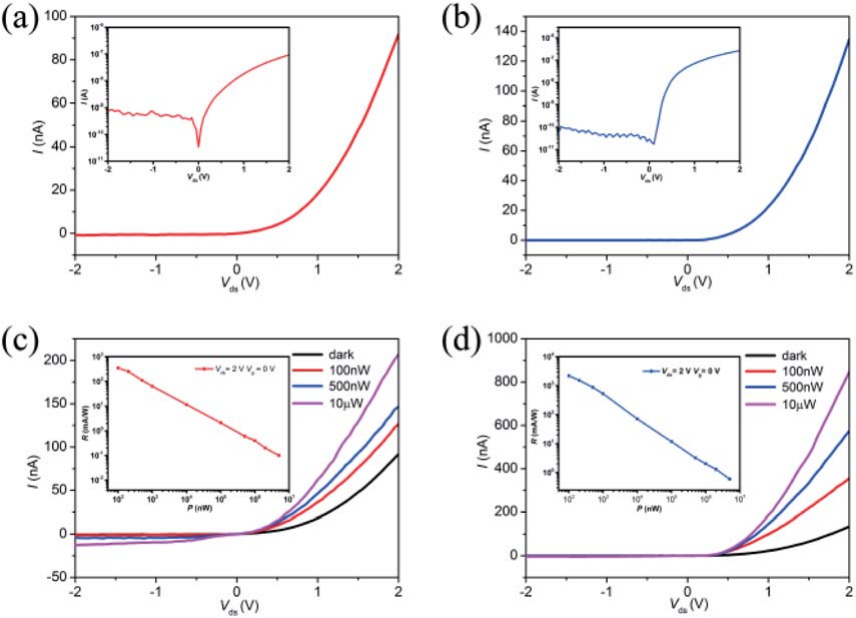
*I*–*V* curves from the left and right BP/MoS_2_ p–n junctions in the SBJT. *I*–*V* curves from the (a) left and (b) right BP/MoS_2_ junctions. The insets show *I*–*V* curves on a log scale. *I*–*V* curves from the (c) left and (d) right BP/MoS_2_ junctions while illuminated with 532 nm laser light at various incident powers. The insets show the photoresponsivity (*R*) at *V*_ds_ = 2 V.

Next, we investigated the electrical characteristics of the SBJT. All the electrical and optoelectrical measurements were performed at room temperature and in ambient conditions within several hours. First, the basis to ensure the BJT's performances is the two p–n junctions between the emitter-base and collector-base. *I*–*V* curves from the two p–n junctions (*i.e.*, the left and right BP/MoS_2_ regions) of the SBJT are shown in Fig. 3a and b, respectively. The insets show these *I*–*V* curves on a log scale. The forward current could be ∼10^−7^ A, while the reverse current is very small, ∼10^−10^ A. The p–n junctions exhibit rectification ratios of ∼127 and ∼4760 at *V*_ds_ = −2 and +2 V, respectively. The ideality factor was calculated with the following equation:

1 *I* = *I*_0_(e^*qV*/*nkT*^ − 1),

where *I* is current through the diode, *V* is the voltage across the diode, *I*_0_ is the dark saturation current, *n* is the ideality factor, *k* is the Boltzmann constant, and *T* is the absolute temperature.^48^ The ideality factors for the left and right BP/MoS_2_ diodes were calculated to be 2.18 and 1.76, respectively, which are similar to the values found in previous studies.^25^ These results demonstrate that the two p–n junctions with excellent rectifying behaviors were formed in the BJT. The reason that the right BP/MoS_2_ diode has a lower ideality factor than the left BP/MoS_2_ diode may be due to the smaller contact resistance between the right BP and MoS_2_ samples. Fig. 3c and d show *I*–*V* curves from the left and right p–n junctions in the SBJT while illuminated with light from a 532 nm laser with various incident powers (the insets show the photoresponsivity (*R*) at *V*_ds_ = 2 V), respectively. The photocurrent increases as the incident light power increases. The photoresponsivity *R* is defined as *I*_ph_/*P*_laser_, where *I*_ph_ is defined as *I*_illumination_–*I*_dark_, and *I*_illumination_ and *I*_dark_ are respectively *I*_ds_ with and without illumination, and *P*_laser_ is the incident laser power. Every p–n junction exhibits *R* values up to 348 mA W^−1^ and 2.2 A W^−1^, respectively. Fig. S4† shows *I*–*V* curves from the left BP/MoS_2_ p–n junction under illumination by a 532 nm laser with various incident powers and top gate bias, illustrating its tunable photodetectivity, which will be discussed later.

**Fig. 4:**
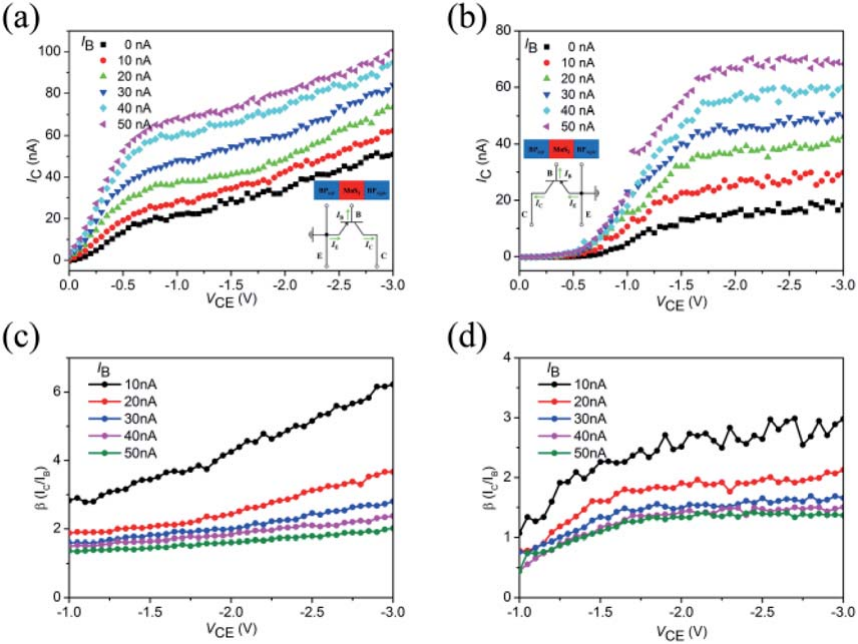
*I*–*V* curves of the SBJT under common-emitter configuration. (a) *I*_C_–*V*_CE_ characterizations at various injection currents (*I*_B_). Inset: the common-emitter configuration, *i.e.* the left BP acts as the emitter (ground), MoS_2_ acts as the base, and the right BP acts as the collector. (b) *I*_C_–*V*_CE_ characterizations at various injection currents (*I*_B_). Inset: the common-emitter configuration, *i.e.* the left BP acts as the collector, MoS_2_ acts as the base, and the right BP acts as the emitter (ground). (c) and (d) The common-emitter current gain (*β* = *I*_C_/*I*_B_) *versus* collector–emitter voltage (*V*_CE_) curves at various injection currents (*I*_B_) corresponding to (a) and (b), respectively.

Under the common-emitter configuration, this SBJT exhibits bidirectional electrical output thanks to its symmetric energy band structure since the emitter and collector have the same thickness and crystal orientation, as shown in Fig. 4. Fig. 4a demonstrates the *I*_C_–*V*_CE_ characterizations of the SBJT at various injection currents (*I*_B_). The inset shows the common-emitter configuration, *i.e.* the left BP acts as the emitter (ground), MoS_2_ acts as the base, and the right BP acts as the collector. It is worth mentioning that when the device is operating in the small *V*_CE_, it would be in the saturation region, the base-emitter junction and base-collector junction are both in the forward bias, leading to majority carriers in the emitter being injected into the base, and the current increase with the increase in *V*_CE_. The collector current did not highly depend on the base current. When the *V*_CE_ is large enough, it will be in the forward active region; the curves exhibit a slight ascendant trend, where *I*_C_ increases with *V*_CE_. Similar results can be seen when the left BP acts as the collector, MoS_2_ acts as the base, and the right BP acts as the emitter, as shown in Fig. 4b. In the saturation region, the current of the collector would not be controlled by the current of the base-emitter junction. It is worth noting that when *V*_CE_ increases to ∼−1.8 V, it would be in the forward active region, and the base-collector junction would transform to reverse bias. The current of the collector *I*_C_ would remain almost constant, and only has a relationship with the injection current (*I*_B_). These results indicate that our device works under ideal conditions, as shown in Fig. 4b. The common-emitter current gain can be defined as *β* = *I*_C_/*I*_B_. The common-emitter current gain *β versus* the collector–emitter voltage (*V*_CE_) curves at various injection currents (*I*_B_) corresponding to Fig. 4a and b are demonstrated in Fig. 4c and d, respectively. The device shows good current gain behaviors and the maximum *β* were calculated to be ∼6 and ∼3, respectively. The current gain *β* of ∼6 is not too big but it is comparable to previously reported results.^32,34,35^ There are two main reasons for the relatively low current gain. First, the gap size made by laser processing between the emitter and collector may be still large. Second, the doping concentration of the carrier between the p-type BP and n-type MoS_2_ may not be properly matched. We believe that the performance could be further improved by optimizing the construction of the device, such as shortening the gap width between the emitter and collector. As the gap width becomes narrow, the transit time of the minority hole in the MoS_2_ (base) decreases, and the probabilities of the recombination of the minority holes and majority electrons are also reduced. As a result, the current gain increases and the device shows better performance. However, there needs to be further investigation on related experiments. We noticed that when the left BP acts as the emitter, the output amplification abilities are better as compared to when the right BP acts as the emitter, which may also be attributed to the smaller contact resistance between the right BP and MoS_2_ samples. To verify the stability of the devices, we performed similar electrical output measurements after two months as shown in Fig. S5.† During this time, we kept this device in a glove box filled with N_2_. We observed that the two p–n junctions still showed good rectifying behaviors, as demonstrated in Fig. S5a and b.† The bidirectional electrical amplification output characterizations under common-emitter configuration are also exhibited in Fig. S5c and d† and these results indicate that our device has good stability.

Usually, as the doping concentration in the emitter increases, the output current increases.^9–13^ Therefore, we placed a top gate on one side of the SBJT to further investigate the effect of electrostatic doping on the device's performance. In our device, the top gate applied on the left black phosphorus (emitter) is a high *κ* hBN dielectric with a thickness of ∼14 nm, therefore, the gate-tunable behavior is more effective and comprehensive. Fig. S6† shows the gate-tunable *I*–*V* curves and corresponding band diagrams when the SBJT is operated in the forward active mode. The common-base configuration is shown in Fig. S6a.† The left BP acts as the emitter, MoS_2_ acts as the base (ground), and the right BP acts as the collector. The top gate was placed on the left BP (emitter). We observed that the collector current *I*_C_ decreased regardless of the gate voltage decrease or increase (Fig. S6b and c†), which is not consistent with our expectations. Fig. S6d–f† shows a schematic and band diagrams of the gate-tunable SBJT in the forward active mode with zero, negative, and positive gate modulation, respectively. We can explain this kind of current transport in the SBJT with the energy band models. Under negative gate modulation, although the concentration of holes in the emitter increases, the barrier's height between the emitter/base increases and thus few holes can cross the left BP/MoS_2_ junction and be collected by the right BP, and the output current decreases. Under positive gate modulation, the height of the barrier decreases. However, the concentration of holes in the emitter decreases and thus few holes can cross the left BP/MoS_2_ junction and be collected by the right BP, yielding a smaller current. More details are presented in the ESI (Fig. S6†). Similar results were obtained in another SBJT. Fig. S7a† shows an optical microscope image of another SBJT. Fig. S7b and c† show *I*–*V* curves from the left and right BP/MoS_2_ p–n junctions in another SBJT (the insets show *I*–*V* curves on a log scale), respectively. Typical rectifying behaviors in the p–n junction were observed at the two junctions with rectification ratios of ∼35 and ∼37 at *V*_ds_ = −2 and +2 V, respectively. For another SBJT, the left BP acts as the emitter, MoS_2_ acts as the base (ground), and the right BP acts as the collector, as was the case in the SBJT discussed in the ESI.† Fig. S8† shows the gate-tunable *I*–*V* curves of this second SBJT. The output current decreases when the gate is positively or negatively modulated. Therefore, there is something meaningful in the collector current phenomenon *I*_C_ and we can conclude that the top gate on the emitter affects the carrier concentration in the emitter and modulates the band alignment between the emitter and base. To get better performance, optimized devices and experiments should be implemented.

**Fig. 5:**
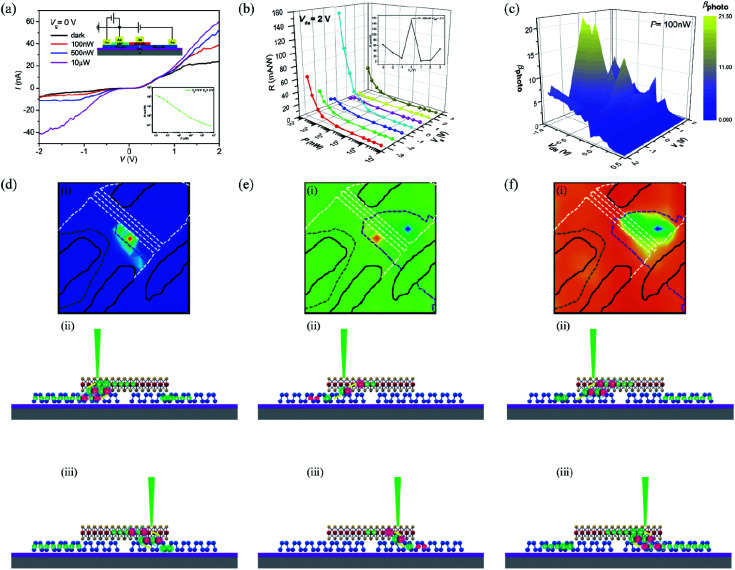
Photoelectric properties of the gate-tunable phototransistor. (a) *I*–*V* curves from the gate-tunable phototransistor while illuminated with 532 nm laser light at various incident powers and *V*_g_ = 0 V. Inset: schematic of the gate-tunable phototransistor and photoresponsivity (*R*) of the device at various incident laser powers with *V*_ds_ = 2 V. *R* = 151 mA W^−1^ at *P* = 100 nW. (b) Gate-tunable *R* with various incident powers at *V*_ds_ = 2 V. The maximum photoresponsivity value is *R* ∼151 mA W^−1^ at *V*_g_ = 0 V. (c) Gate-tunable optical gain *β* with varying *V*_ds_ at *P* = 100 nW. The maximum optical gain is *β* = 21 at *V*_g_ = 0 V. Scanning photocurrent images at different biases with zero gate voltage and corresponding schematic diagram of the device illuminated with 532 nm laser light; (d) *V*_ds_ = 2 V, (e) *V*_ds_ = 0 V, and (f) *V*_ds_ = −2 V.

Our SBJT can also operate as a phototransistor with gate-tunable photocurrent amplification when the base is left floating. Two important parameters for evaluating a phototransistor are its photoresponsivity (*R*) and gain (*β*).^42^*β* is defined as *β*_photo_ = *I*_ds_/*I*_pn_ for a given level of illumination,^33,42^ when the base is left floating; *I*_ds_ is the two-terminal current measured between the left and right BP regions. The gain here is in reference to the photocurrent generated in the left BP/MoS_2_ p–n diode (*I*_pn_), which we measured previously. The gate-tunable photoelectric properties of the phototransistor are shown in Fig. 5. Fig. 5a shows *I*–*V* curves of the phototransistor illumination with a 532 nm laser at various incident powers with *V*_g_ = 0 V. The photocurrent increases as the input illumination increases, which is attributed to the increased photoinduced carrier concentration. The insets show a schematic of the gate-tunable phototransistor and the photoresponsivity (*R*) of the device at various incident laser powers with *V*_ds_ = 2 V and *V*_g_ = 0 V. The value *R* = 151 mA W^−1^ was calculated at *P* = 100 nW. The gate-tunable *I*–*V* curves from the illuminated phototransistor can be seen in Fig. S9.†Fig. 5b shows the gate-tunable photoresponsivity *R* at various incident powers with *V*_ds_ = 2 V; a maximum photoresponsivity of *R* = 151 mA W^−1^ was observed at zero gate bias. Similar to the case demonstrated in Fig. S6,† the photocurrent decreases when the gate voltages decrease due to higher barrier at the emitter-base junction, while increasing the gate voltage decreases the holes' concentration, which also decreases the photocurrent. The gate-tunable optical gain *β* with varying *V*_ds_ is shown in Fig. 5c, and maximum gain of *β* ∼ 21 was found at *V*_g_ = 0 V; although this result is not very outstanding, it is better than that of other bipolar phototransistors,^40,42,49,50^ as shown in Table 1. The maximum *β* value was also observed at zero gate bias for the same reason stated above. Scanning photocurrent images (SPI) were gathered at zero gate bias and while the device was illuminated with 532 nm laser light in order to determine the mechanism governing the photocurrent amplification. Fig. 5d shows the SPI and corresponding schematic diagram of the device at *V*_ds_ = 2 V. Apparent photocurrent signals can be observed at the interface between the left BP region and MoS_2_, as shown in Fig. 5d(i). Under forward bias, holes were driven from the left BP region to right BP region by the electric field, forming a forward current. When the left BP/MoS_2_ interface region was illuminated, photoinduced electron–hole pairs will be separated by the electric field at the junction, and holes flow into the base and increase the photocurrent, as shown in Fig. 5d(ii). On the other hand, although there are photogenerated electron–hole pairs at the right BP/MoS_2_ junction when this region is excited, the separated electrons will recombine with holes from the base, which does not affect photocurrent amplification, as shown in Fig. 5d(iii). Fig. 5e shows the SPI and corresponding schematic diagram of the device at zero bias. Due to the photovoltaic effect, the photogenerated electron–hole pairs will separate into free charges at the p–n interface and form a photocurrent driven by the built-in electric field. The two p–n junctions have mirror symmetry and thus, two opposite photocurrents can be observed at the left and right BP/MoS_2_ interface regions. Fig. 5f(i) shows the SPI from the device at *V*_ds_ = −2 V. One can see that the strongest photoresponse occurs at the right BP/MoS_2_ interface, which is attributed to photoinduced carriers at the junction. The symmetric structure of our phototransistor can be explained as illustrated in Fig. 5d. The photogenerated electron–hole pairs will be separated by the electric field at the right BP/MoS_2_ junction when this region is excited; holes move into the base and amplify the backward photocurrent, as shown in Fig. 5f(ii). When the left BP/MoS_2_ interface region is illuminated, photoinduced electron–hole pairs will be separated by the electric field at the junction; separated electrons will recombine with holes in the base, leading to very low photocurrent (Fig. 5f(iii)). The gate-tunable SPI from the phototransistor are shown in Fig. S10.† In spite of the various gate bias values, the photocurrent is primarily concentrated in the same location for a given bias. The strongest photocurrent was measured when the gate was held at 0 V, and the photoresponse decreased gradually when the gate bias was non-zero. These phenomena also occur due to the broken symmetry at non-zero gate bias.

**Table 1:** Comparison of photocurrent gains in this work and other previously reported phototransistors devices

Material	Type	Structure	*β*	Ref.
Au/graphene/MoS_2_	NPN	3D	∼18	49
Si/Ge/Si	NPN	3D	∼7	40
MoS_2_/BP/WSe_2_	NPN	2D	∼9.8	42
MoS_2_	NPN	2D	∼23	50
BP/MoS_2_/BP	PNP	2D	∼21	This work

## Conclusions

In summary, we presented a symmetric PNP SBJT fabricated from p-type BP and n-type MoS_2_ with FSLP. Compared with other intricate growth procedures and tedious transfer processes, we can produce BJT with a single stacking step with the advantage of femtosecond laser processing. On this basis, femtosecond laser processing allows us to reduce the cost and time of the fabrication process, improve the success rate of the device fabrication. This SBJT exhibits a rectification ratio of 10^3^ and photoresponsivity of 2.2 A W^−1^. This SBJT exhibits bi-directional electrical output due to its symmetric band structure since the emitter and collector have the same thickness and crystal orientation. Moreover, this SBJT can act as a kind of phototransistor with maximum photoresponsivity of *R* = 151 mA W^−1^ and maximum photocurrent gain of *β* ∼ 21. Better performance could be further achieved by optimizing the construction of the device, such as shortening the gap width between the emitter and collector. Scanning photocurrent images (SPI) were used to determine the mechanism governing photocurrent amplification in the SBJT. These results illustrate a novel, convenient method for fabricating multifunctional heterostructure devices.

## Experimental section

### Fabrication of symmetric bipolar junction transistor

Black phosphorus thin flakes of uniform thickness were mechanically exfoliated using adhesive tape (3M Scotch) from bulk BP crystals (XFNANO, Inc) on a clean SiO_2_/Si substrate. The thickness of SiO_2_ on the p-doped Si substrate was ∼285 nm. FSLP (800 nm, 35 fs, and 30 mW) was used to cut the BP flake into two pieces. More details regarding FSLP are given in Fig. S1.† A MoS_2_ flake was bridged onto the two BP pieces using a dry-transfer technique with a micromanipulator and an optical microscope. Subsequently, an hBN flake was transferred onto the left BP flake as the top gate dielectric in the same way. Afterwards, 50 nm Au electrodes were patterned using photolithography with the femtosecond laser (800 nm, 35 fs) and deposited using magnetron sputtering (JZCK-465 of Sky Technology Development). Fig. S2† shows the corresponding optical microscope images of the SBJT after each fabrication step. Finally, vacuum thermal annealing (320 °C for 1 h) was performed to achieve good contact.

### Characterizations

Raman spectroscopy and AFM were measured using a confocal Raman/AFM system (Alpha 300 R, WITec). Raman spectroscopy was conducted using a 532 nm laser with power of 3 mW (integration time of 30 s and number of accumulations of 5), and the resolution of the fine Raman spectrum was above 0.1 cm^−1^.

### Electrical and optoelectrical measurements

All the electrical and photoresponse measurements were gathered under ambient conditions and at room temperature. The electrical and optoelectronic characteristics were measured with a semiconductor parameter analyzer (Keithley 4200A), and the SPI were gathered with digital source-measure units (Keithley 2400 and 2450).

## Conflicts of interest

There are no conflicts to declare.

## Supplementary Material

NA-002-D0NA00201A-s001
