# Supplementary material for: A gate-tunable symmetric bipolar junction transistor fabricated via femtosecond laser processing
Source: Nanoscale Adv. 2020 Mar 18;2(4):1733–40. doi: 10.1039/d0na00201a (PMC9417257; doi:10.1039/d0na00201a)
Supplement: NA-002-D0NA00201A-s001 [file NA-002-D0NA00201A-s001.pdf]

Electronic Supplementary Material (ESI) for Nanoscale Advances.
This journal is © The Royal Society of Chemistry 2020

Supporting Information

Gate Tunable Symmetric Bipolar Junction Transistor Fabricated via Femtosecond Laser Processing

*Bao-Wang Su[a], Bin-Wei Yao[b], Xi-Lin Zhang[a], Kai-Xuan Huang[a], De-Kang Li[a], Hao-Wei Guo[a], Xiao-Kuan Li[a], Xu-Dong Chen[b], Zhi-Bo Liu[*a,c,d], Jian-Guo Tian[a,c,d]*

[a]The The Key Laboratory of Weak Light Nonlinear Photonics, Ministry of Education, Teda Applied Physics Institute and School of Physics, Nankai University, Tianjin 300071, China. E-mail:liuzb@nankai.edu.cn

[b]Institute for New Energy Materials and Low Carbon Technologies, School of Materials Science and Engineering, Tianjin University of Technology, Tianjin 30071, China

[c]Renewable Energy Conversion and Storage Center, Nankai University, Tianjin 300071, China

[d]The collaborative Innovation Center of Extreme Optics, Shanxi University, Taiyuan, Shanxi 030006, China

Corresponding Author

*****E-mail: liuzb@nankai.edu.cn

S1. The procedure for the optical microscope images of the femtosecond laser processing (FSLP).

In our works, the femtosecond laser pulses at central wavelengths of 800 nm with a pulse width of 35 fs were focused by a 100 × objective len with a numerical aperture of 0.9 onto the black phosphorus (BP) flake surface. The incident laser power was 30 mW.

Figure S1 shows the procedure for the optical microscope images of the femtosecond laser processing (FSLP). Figure S1a presents the optical microscope image of the pristine BP flake. Figure S1b exhibits the optical microscope image of the BP flake after femtosecond laser processing. In this process, the illuminated area of BP (as the white arrows indicate) would be oxidized and vaporized rapidly when the incident light spot focused the BP sample. The oxidized phosphorus could be further cleaned up after washing in the deionized water, as demonstrated in Figure S1c.

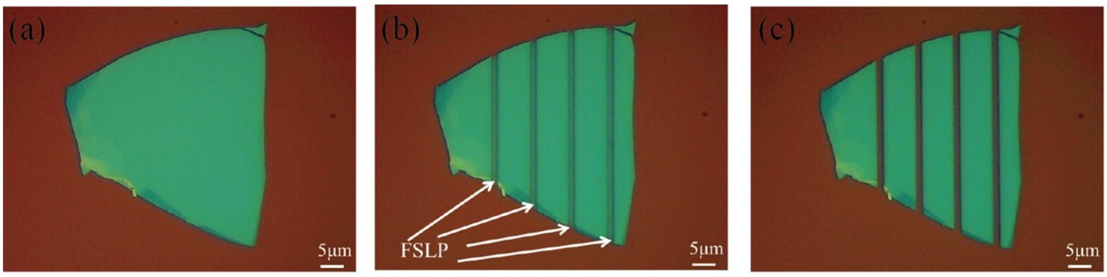

Figure S1. The procedure for the optical microscope images of the femtosecond laser processing (FSLP). (a) The pristine BP flake. (b) The BP flake after FSLP. (c) The BP flake after being washed in the deionized water.

S2. The optical microscope images of the fabrication procedure for the BP/MoS_2/BP SBJT.

Figure S2 shows the corresponding optical microscope images of the SBJT after each fabrication step. First, BP thin flake of uniform thickness was mechanically exfoliated using adhesive tape from a bulk BP crystal on a 285 nm SiO_2/p+-doped Si substrate, as shown in Figure S2a. And then, we use FSLP (800 nm, 35 fs, and 30 mW) to cut it into two separate BP pieces, as demonstrated in Figure S2b. Next, MoS_2 flake was bridged onto the two separate BP pieces by dry-transfer technique (Figure S2c). Subsequently, hBN flake as the top gate dielectric was transferred onto the left BP flake with the same way (Figure S2d). Finally, 50 nm-thick Au as metal electrodes were patterned and deposited using photolithography and magnetron sputtering (Figure S2e).

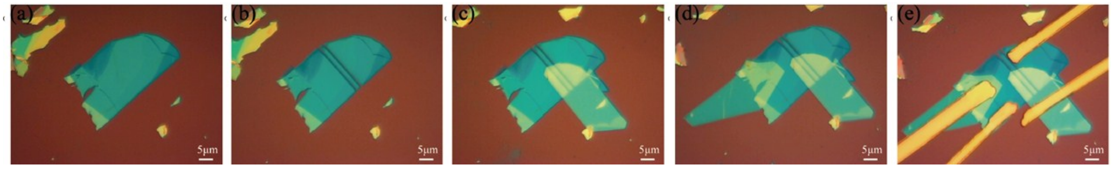

Figure S2. The optical microscope images of the fabrication procedure for the BP/MoS_2/BP SBJT. (a) The pristine BP flake. (b) The BP flake after FSLP. (c) Transfer MoS_2 flake. (d) Transfer hBN flake. (e) Deposite Au metal electrodes.

S3. The AFM images and height profiles of device components.

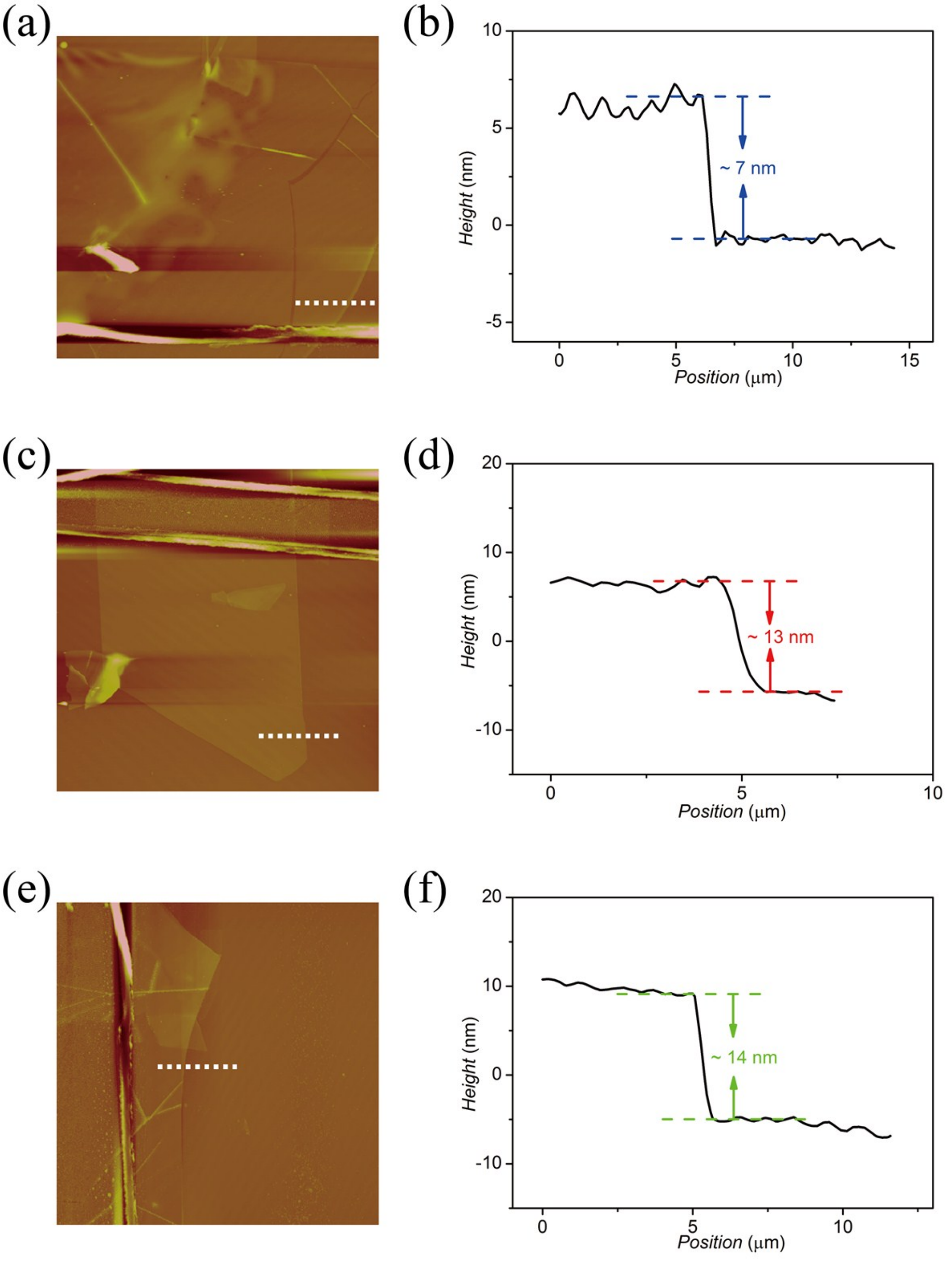

Figure S3. The AFM images and height profiles of device components. (a), (c) and (e) shows BP flake, MoS_2 flake and hBN flake, respectively. (b), (d) and (f) shows the corresponding thickness ~7 nm, 13 nm and 14 nm, respectively.

S4. *I−V* characteristics of the left BP/MoS_2 p-n junction under illumination by 532 nm laser with various incident powers and gate bias.

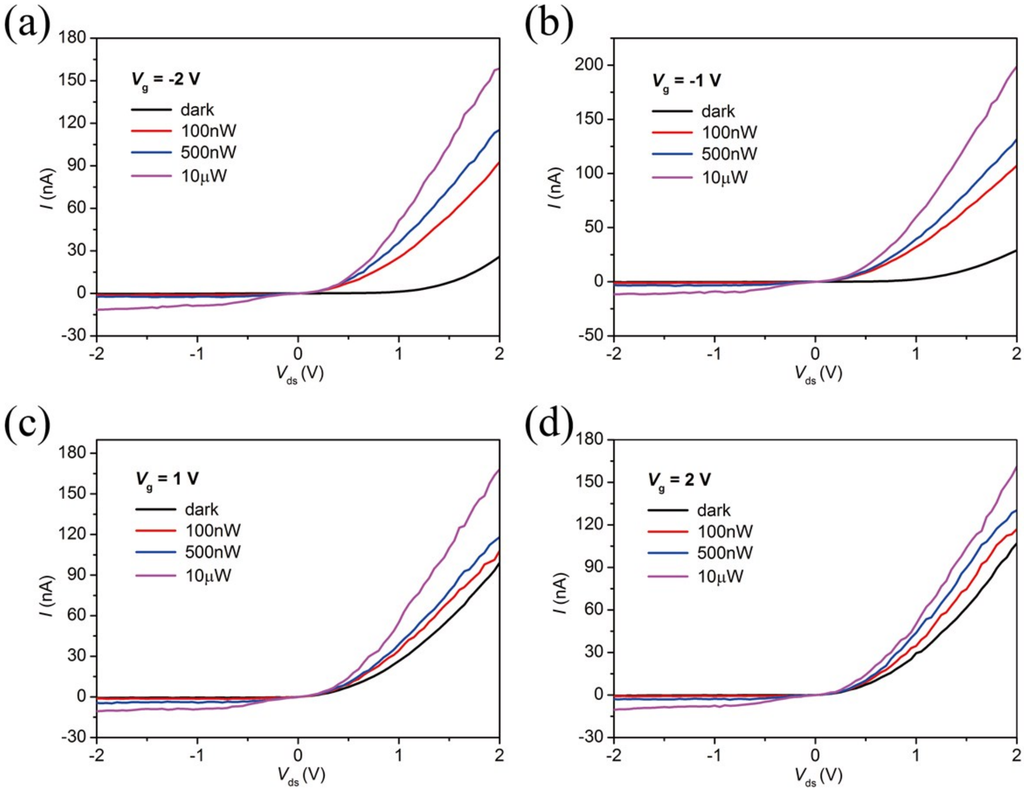

Figure S4. *I−V* characteristics of the left BP/MoS_2 p-n junction under illumination by 532 nm laser with various incident powers and gate bias. (a) V_g = -2 V. (b) V_g = -1 V. (c) V_g = 1 V. (d) V_g = 2 V.

S5. $I-V$ characteristics of the device after two month.

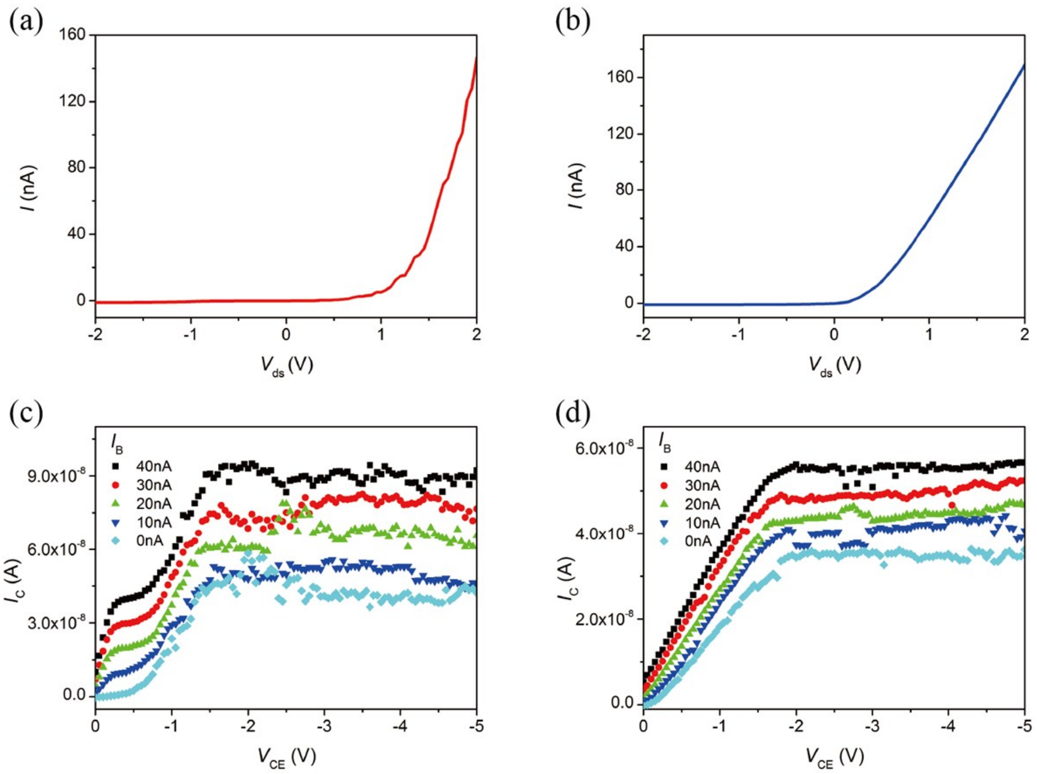

Figure S5. $I-V$ characteristics of the device after two month. (a) left BP/MoS_2 junction. (b) right BP/MoS_2 junction. (c) I_C-V_{CE} characterizations under common-emitter configuration i.e. the left BP acts as the emitter (ground), MoS_2 acts as the base, and the right BP acts as the collector. (d) I_C-V_{CE} characterizations under common-emitter configuration i.e. the left BP acts as the collector, MoS_2 acts as the base, and the right BP acts as the emitter (ground).

S6. Gate tunable *I-V* curves and corresponding band diagrams when the SBJT is operated in forward active mode.

Figure S6 shows the gate tunable *I-V* curves and corresponding band diagrams when the SBJT is operated in forward active mode. The common-base configuration is shown in Figure S6a. The left BP acts as the emitter, MoS_2 acts as the base (ground), and the right BP acts as the collector. The top gate was placed on the left BP (emitter). Figure S6b shows *I-V* curves of the gate tunable SBJT with a negative gate voltage V_g at $I_B = 0$ A. One can see that the output current I_C decreases as V_g decreases. *I-V* curves under positive gate V_g at $I_B = 0$ A are shown in Figure S6c. One should note that the output current I_C decreases as V_g increases. Figure S6d-f shows a schematic and band diagrams of the gate tunable SBJT in forward active mode with zero, negative, and positive gate modulation, respectively. And we can explain this kind of current transport in the SBJT with these energy band models. When the SBJT was operated in forward active mode, i.e., the base-emitter (MoS_2-left BP) was forward biased and the base-collector (MoS_2-right BP) was reverse biased. Holes are the dominant charge carrier in the PNP BJTs. Under zero gate voltage, holes in the emitter overcome the barrier to cross the left BP/MoS_2 junction and are drawn to the collector by the electric field in the right BP/MoS_2 junction, producing the output current. Under negative gate modulation, the concentration of holes in the emitter increase, i.e. the doping concentration increase. As a result, the depletion region between the emitter and base becomes wider. As the depletion region becomes wider, the built-in electric field increases, resulting in the increases of height of the barrier. On the other hand, as the increase of doping concentration, the Fermi level in the emitter will shift downward towards the valence band. However, the Fermi level in the base keeps still, thus, the difference of the Fermi level between the emitter and base increases, also leading to the increases of height of the barrier. Although the concentration of holes in the emitter increases, the barrier's height between the emitter/base increases, thus few holes can cross the left BP/MoS_2 junction and be collected by the right BP, and the output current decreases. Under positive gate modulation, the doping concentration decrease, and a narrower depletion region will be formed between the emitter and base, leading to the height of the barrier decrease. On the other hand, as the doping concentration decrease, the Fermi level in the emitter is shifted toward to the conduction band. Considering the Fermi level in the base keeps fixed, therefore, the difference of the Fermi level between the emitter and base decreases, also

leading to the decreases of height of the barrier. However, the concentration of holes in emitter decreases as was as under positive gate modulation, thus few holes can cross the left BP/MoS_2 junction and be collected by the right BP, yielding a smaller current.

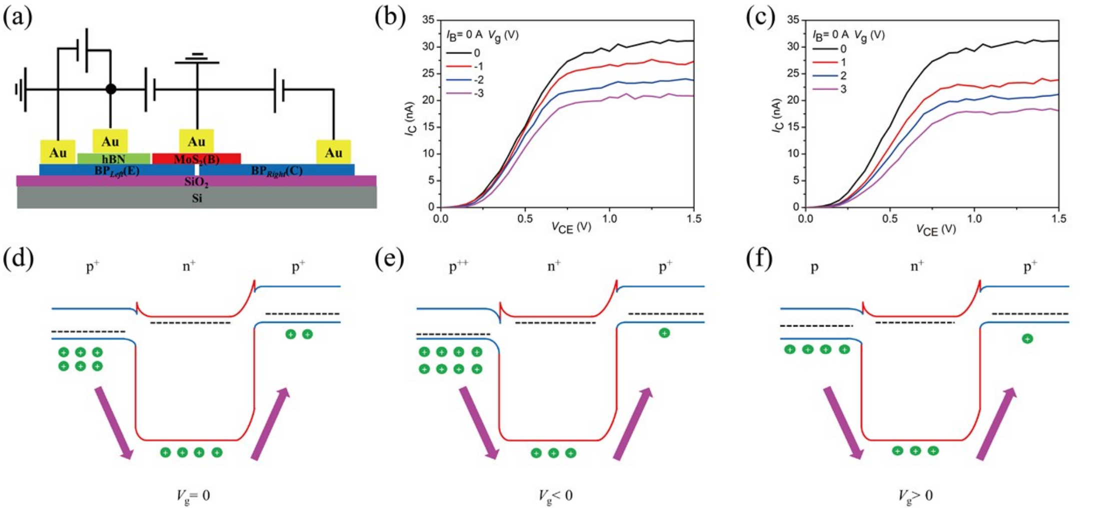

Figure S6. Gate tunable *I-V* curves and corresponding band diagrams in the SBJT. (a) Schematic showing the common-base configuration of the gate tunable SBJT. The left BP acts as the emitter, MoS_2 acts as the base (ground), and the right BP acts as the collector. The top gate was placed on the left BP (emitter). (b) *I-V* curves from the gate tunable SBJT with negative gate voltage V_g at I_B = 0 A. (c) *I-V* curves from the gate tunable SBJT with positive gate voltage V_g at I_B = 0 A. Schematic and band diagrams of the gate tunable SBJT at (d) zero, (e) negative, and (f) positive gate modulation.

S7. *I-V* characteristics of the two p-n junctions (i.e. the left BP/MoS_2 and the right BP/MoS_2) of another SBJT.

Figure S7a shows the optical microscope image of another SBJT. Blue outlines the left BP and right BP, red outlines the MoS_2 and green outlines the hBN, respectively. The *I-V* characteristics of the two p-n junctions (i.e. the left BP/MoS_2 and the right BP/MoS_2) of another SBJT were demonstrated in Figure S7b and Figure S7c (the insets show the *I–V* characteristics on a log scale), respectively. Typical rectifying behaviors of the p-n junction were both observed at the two junctions, and the rectification ratios of ~ 35 and ~ 37 at V_{ds} = -2/+2 V, respectively.

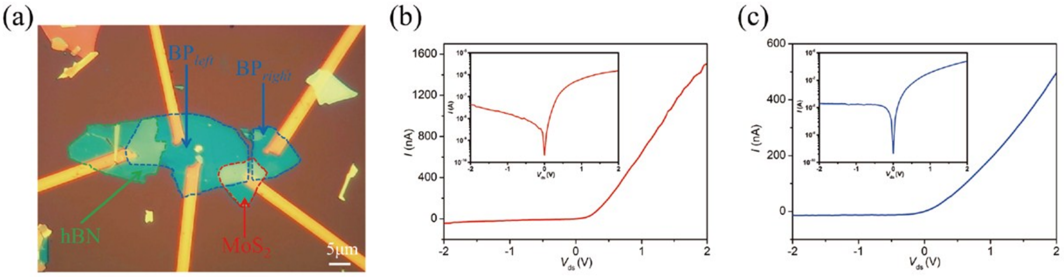

Figure S7. *I-V* characteristics of the two p-n junctions (i.e. the left BP/MoS_2 and the right BP/MoS_2) of another SBJT. (a) The optical microscope image of another SBJT. Blue outlines the left BP and right BP, red outlines the MoS_2 and green outlines the hBN, respectively. *I-V* characteristics of (b) left BP/MoS_2 junction (c) right BP/MoS_2 junction. The insets show the *I–V* characteristics on a log scale.

S8. Gate tunable *I-V* characteristics of another SBJT.

Figure S8 demonstrates the gate tunable *I-V* characteristics of another SBJT. No matter under negative gate modulation (Figure S8a) or positive gate modulation (Figure S8b), the output current decreases.

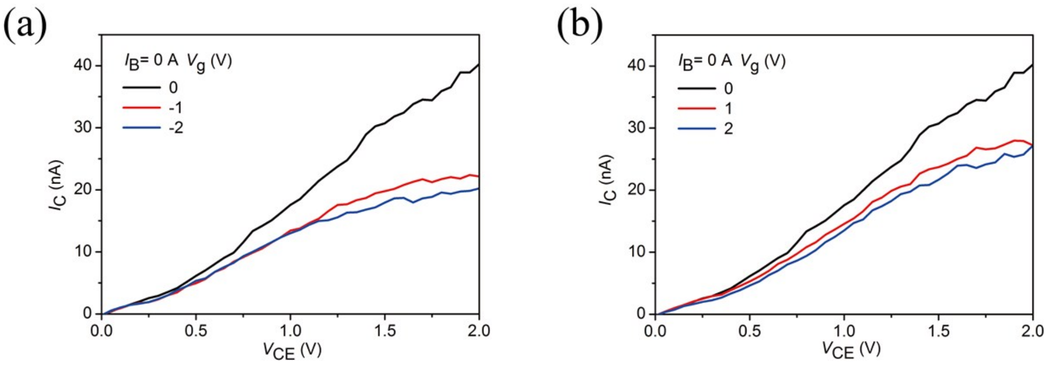

Figure S8. Gate tunable *I-V* characteristics of another SBJT. (a) *I-V* characteristics of the gate tunable SBJT under negative gate V_g at $I_B = 0$ A. (b) *I-V* characteristics of the gate tunable SBJT under positive gate V_g at $I_B = 0$ A.

S9. *I−V* characteristics of the gate tunable phototransistor under illumination by 532 nm laser with various incident powers and gate bias.

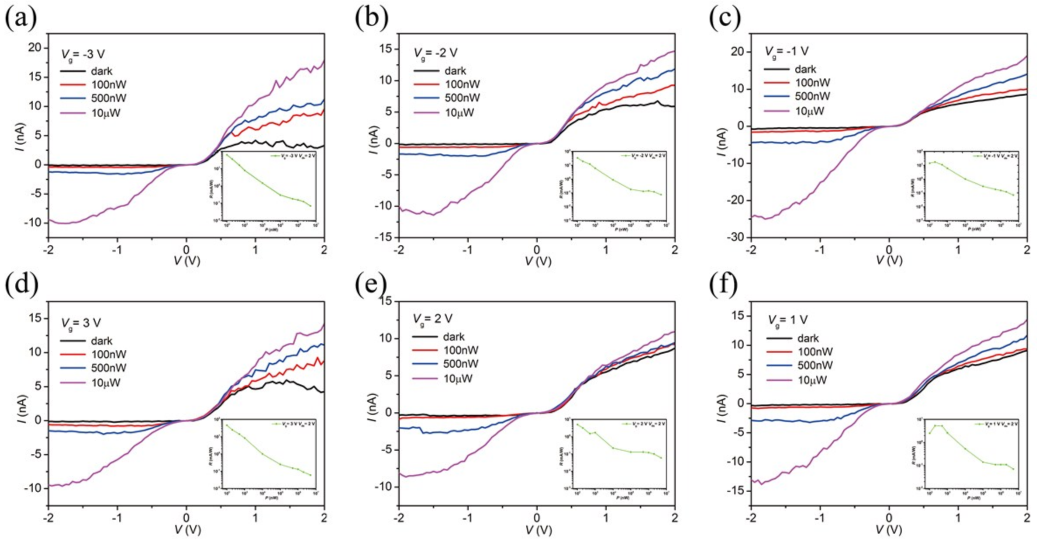

Figure S9. *I−V* characteristics of the gate tunable phototransistor under illumination by 532 nm laser with various incident powers and gate bias. Inset: photoresponsivity (*R*) of the device under various incident laser powers while V_{ds} = 2 V. (a) V_g = -3 V. (b) V_g = -2 V. (c) V_g = -1 V. (d) V_g = 3 V. (e) V_g = 2 V. (f) V_g = 1 V.

S10. Gate tunable scanning photocurrent images of the phototransistor under various bias.

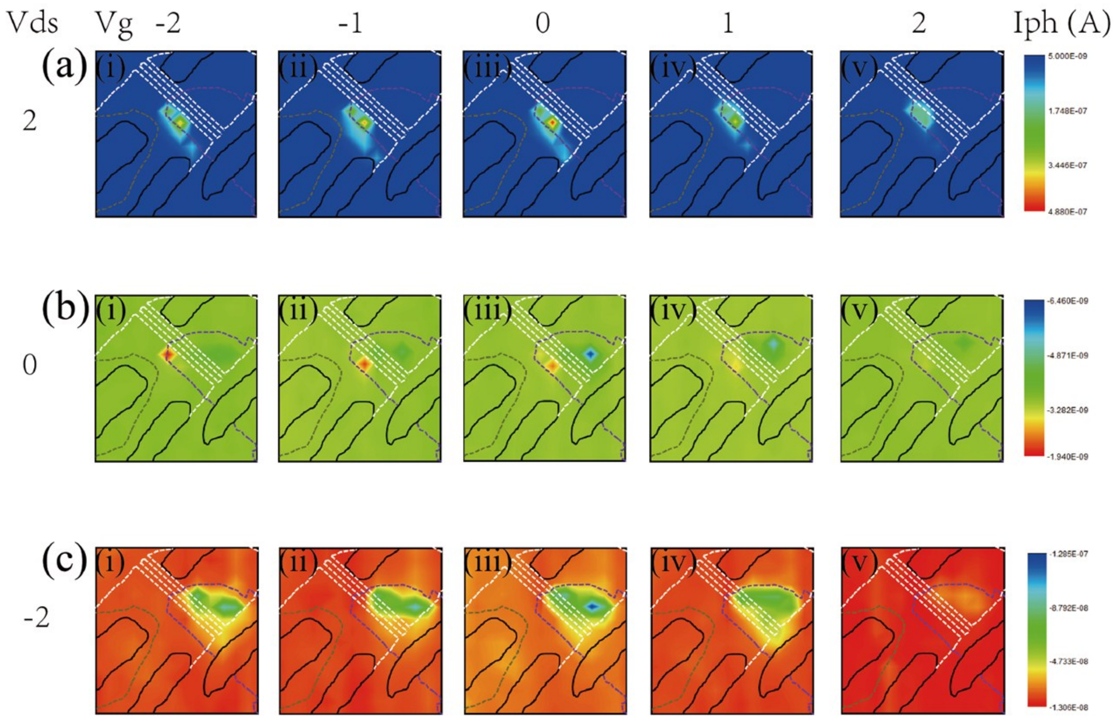

Figure S10. Gate tunable scanning photocurrent images of the phototransistor under (a) forward bias, (b) zero bias and (c) backward bias, respectively.
